# Opacification Kinetics of PLA during Liquid Water Sorption

**DOI:** 10.3390/polym16182621

**Published:** 2024-09-16

**Authors:** Sara Liparoti, Roberto Pantani

**Affiliations:** Department of Industrial Engineering, University of Salerno, Via Giovanni Paolo II, 132, 84084 Fisciano, Italy; sliparoti@unisa.it

**Keywords:** PLA, opacification, water sorption, diffusion

## Abstract

When in contact with water, poly(lactic acid), PLA, undergoes several physical changes. A very evident one is opacification, namely the change from the typical transparent appearance to a white opaque color. This phenomenon is particularly significant for many applications, including packaging, since opacity hinders the possibility of a clear look of the packed goods and also worsens the consumers’ perceptions. In this work, we report an analysis of the time evolution of the phenomenon in different conditions of temperature and water concentration. The results allow us to define a time-scale of the phenomenon and to put it in relationship with the temperature and water content inside the material. In particular, opacification proceeds from the outer surface of the specimens toward the center. Both craze formation due to hydrolysis and crystallization contribute to the opacification phenomenon. Opacification becomes faster as temperature increases, whereas the increase in the solution density has the opposite effect. A model for describing the evolution of opacification was proposed and found to be consistent with the experimental data.

## 1. Introduction

Poly(lactic acid) (PLA) is a biodegradable and bio-based polymer derived from renewable resources such as corn starch and sugarcane. Its favorable properties, including biodegradability, high strength, and versatility, have led to its widespread adoption in various applications, particularly in packaging [1,2,3,4,5], biomedical devices [6,7,8,9,10], and 3D printing [11,12,13,14,15]. However, one of the challenges limiting the broader use of PLA, especially in long-term applications, is the adsorption of moistures [16,17,18]. Water sorption in PLA can significantly influence its physical, mechanical, and thermomechanical properties [16,19,20,21]. One of the notable effects is the opacification of PLA, a phenomenon where the material transitions from a transparent to an opaque appearance upon exposure to moisture. This change in appearance is a critical factor in applications where optical clarity is essential, such as in packaging films and medical devices.

Previous works [22] have shown that when PLA comes into contact with water, the increase in opacity is the first observable effect, occurring within a timeframe that aligns with water sorption. This opacification is likely caused by the formation of crazes, induced by stresses from swelling at the water penetration front. Other phenomena, such as the evolution of crystallinity and hydrolysis, become evident at longer times. Moisture enters the polymer matrix via the diffusion of the water molecules through the material. Random molecular motion transports water molecules with a rate that depends on temperature and inherent moisture content [23]. Deroine et al. [24] induced accelerated aging in injection-molded PLA parts, adopting distilled water and seawater as moistures in the 25–50 °C range. The test revealed morphological changes and porosity creation due to moisture adsorption in the polymer matrix. Harris et al. [25] monitored crystalline and amorphous PLA samples undergoing moisture adsorption in a humidity chamber maintained at 50 °C and 90% humidity. High temperatures and humidities accelerate the aging phenomenon. Their study proved that the injection-molded PLA grades are unsuitable for applications requiring long-term durability in environments that are subject to elevated temperature and humidity. Rocca-Smith et al. [26] analyzed the effect of the moisture state on PLA aging. They found a strong link between the degradation rate and the state of moisture: degradation in saturated vapor medium proceeds faster than in liquid water. The difference was ascribed to the fact that when the PLA part is immersed in a liquid, moisture degradation products are transferred into the liquid medium. In contrast, in the case of vapor, they remain in the polymer matrix, acting as a catalyst. Demirtaş et al. [27] proved that the mechanical properties in PLA 3D-printed parts decrease as the humidity increases. The presence of fillers in the polymer matrix may delay the phenomenon since they act as a barrier for water diffusion into the matrix. Kakanuro et al. [28] analyzed the effect of SiC loaded in PLA on the adsorption rate and found that the presence of SiC significantly increases the durability of the parts. Plasticizers, generally used to improve the flexibility in PLA [29] packages, are recognized to increase opacity; thus, they are expected to make the opacification phenomenon faster.

Despite the huge efforts devoted to analyzing PLA degradation, only a few works analyze the effects of temperature and moisture density on the appearance of the parts. To the authors’ knowledge, no paper has been devoted to analyzing the evolution of opacification and the effects of temperature and moisture on such kinetics. Understanding the impact of the main physical factors, such as temperature and water concentration, as well as their interplay, is crucial for predicting the long-term performance of PLA in humid environments and for developing strategies to mitigate these effects.

This study aims to investigate the relationship between water sorption and the opacification of PLA. By examining the kinetics of water uptake within the polymer matrix, we seek to provide an understanding of the effects of temperature and water content on opacification. This research will contribute to developing more durable PLA-based materials with enhanced resistance to moisture-induced degradation, thereby expanding the potential applications of this important biopolymer. The modeling part will allow for predicting the evolution of the phenomenon over time.

## 2. Material and Methods

**Figure 1:**
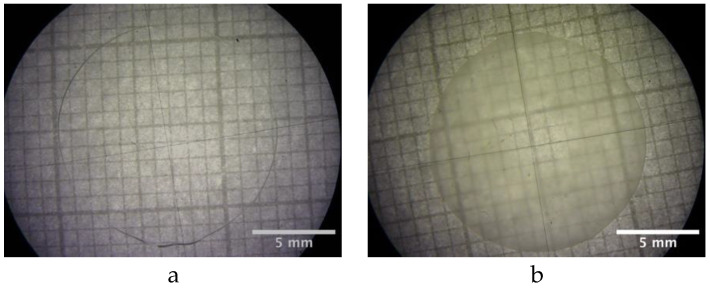
Optical image of the PLA sample (**a**) before and (**b**) after the test at 58 °C in water.

**Figure 2:**
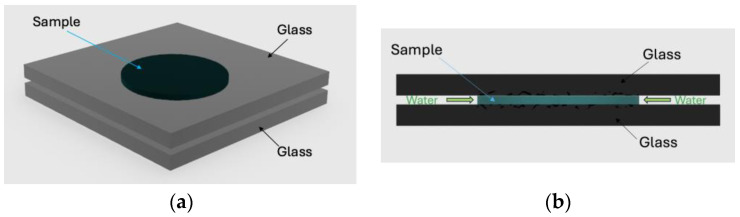
Schematic view of the system adopted for water sorption, composed of two glass slides, each 1 mm thick, and the PLA disc that is 0.4 mm thick, with a diameter of 15 mm. (**a**) 3D view, (**b**) side view. The green arrows show the direction of water sorption.

The PLA adopted in this work is the commercial grade 4032D by Natureworks (Mn = 120 kg/mol, Mw = 210 kg/mol, D-isomer content about 2%). The sample was prepared by melting a previously dried and weighed single pellet between two heated microscope glass slides on a hot plate at 200 °C. In this process, the pellet became a transparent disc with a diameter of about 13 mm and a thickness of 0.4 mm, as shown in Figure 1, stuck on both circular faces on the two glass slides. When immersed in water, the system, composed by the sample and the two glass slides, allows contact between water and PLA only on the lateral surface of the disk, as schematically shown in Figure 2, to ensure water diffusion in the radial direction

**Figure 3:**
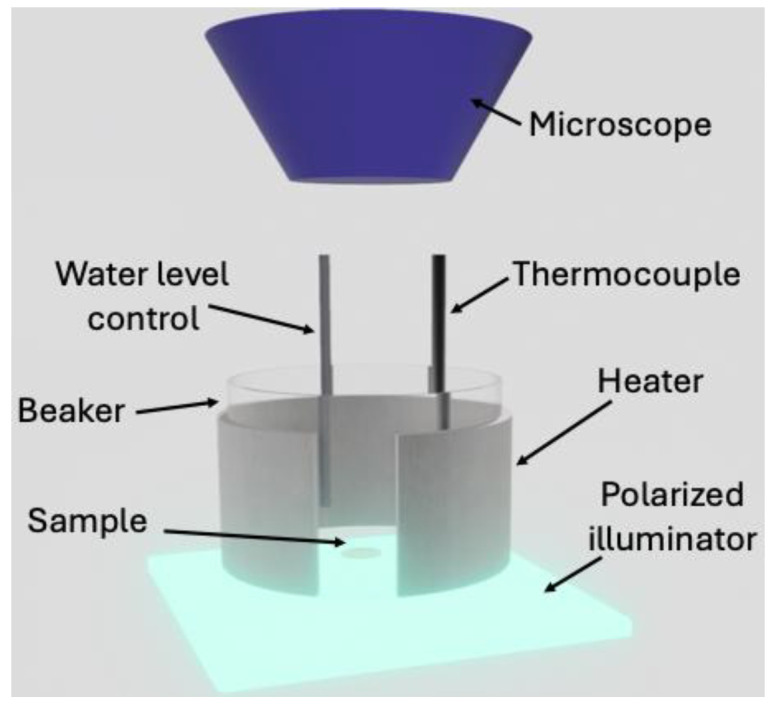
Schematic view of the experiment setup.

The system was placed in a beaker containing distilled water or a solution of water and calcium nitrate with a chosen density, as specified below. Temperatures of 48 °C, 53 °C, and 58 °C were chosen so that the samples were below the glass transition of PLA, which is about 60 °C [30], but above room temperature, at which no significant effect on PLA was evident up to several weeks of immersion in water [31]. The use of solutions of water and calcium nitrate was aimed at reducing the activity of water without changing the pH of the medium, which is known to influence the degradation of PLA [32]. The equilibrium water concentration inside the solid samples is reduced by increasing the salt concentration for the same system temperature. Densities of 1.1 g/cm^3^ and 1.15 g/cm^3^ were chosen. At larger concentrations, the solutions were too cloudy to allow for a clear detection of the phenomenon. A scheme of the system is reported in Figure 3. The level of water and the temperature were continuously controlled; a polarized LED illuminator was placed below the beaker, and a microscope was adopted to capture the phenomenon.

An Olympus BX51 (Olympus Italia, Segrate, Milan) microscope was used to acquire optical micrographs with parallel and cross polarizers. The pictures taken during the tests were analyzed using software for image processing (ImageJ—https://imagej.net URL accessed 1 September 2024). After converting each image to grayscale, the software allows plotting the grayscale level along a given path. The grayscale level was then normalized (from 0 to 1) with respect to the maximum value reached during the test. By considering a threshold equal to 0.5, it was possible to quantify the advancement of the opacification front.

Five different testing conditions were used by changing the water temperature and the density of the solution, as reported in Table 1. The equilibrium water content of the samples, $C_{eq}$, was determined by placing a sample of known dry weight, 0.5 mm thick, in a solution at a given temperature and density for approximately 5 h, namely a time long enough to allow the equilibrium. Afterward, the sample was removed from the solution, dried with a cloth, and weighed. The water content was then calculated and converted to concentration according to the following equation:

(1) $$C_{eq}=\frac{M_{wet}-M_{dry}}{{Mw}_{H_{2}O}}\frac{\rho_{PLA}}{M_{dry}}$$

in which $M_{wet}$ and $M_{dry}$ are the masses of the sample before and after the water immersion, ${Mw}_{H_{2}O}$ is the molecular weight of water, and $\rho_{PLA}$ is the density of PLA, taken as 1250 kg/m^3^ [33].

The results are presented in Table 1. As expected, the water absorbed by the polymer decreases as the salt concentration (i.e., the solution density) increases and the temperature decreases.

For each test condition, at least two samples were analyzed.

## 3. Results

### 3.1. Sample A, Distilled Water at 58 °C

As reported in Figure 1b, the sample appears white and opaque after the test.

**Figure 4:**
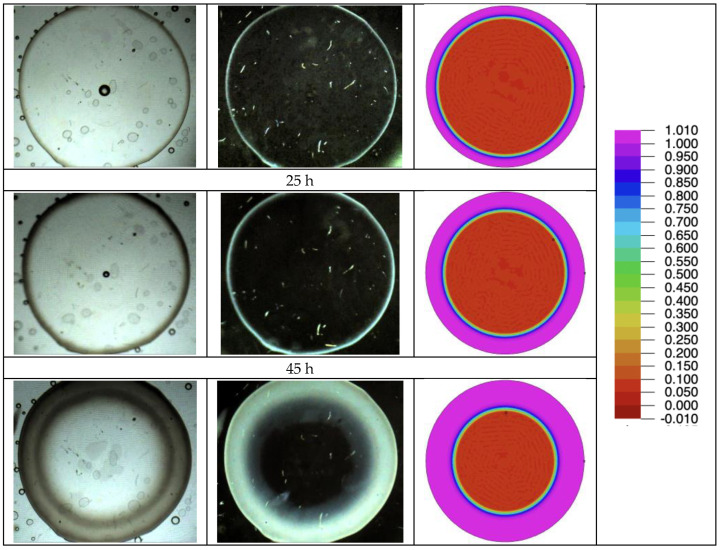

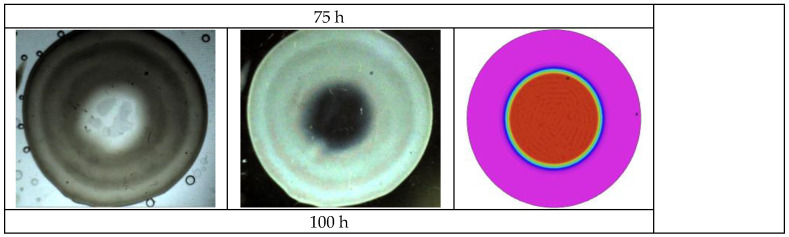
Micrographs of the PLA samples were taken at different times during the test at 58 °C (test A). Left column, optical images; central column, polarized optical images; right column, results of simulation with a color scale (results from Equation (5)): red is transparent, fuchsia is fully opaque, green demarcates the thickness of the opaque ring.

In Figure 4, the time evolution of the appearance of sample A is reported at selected time intervals, both in transmitted light (left column) and crossed polarized light (right column). The opacification of the sample is quite evident and starts from the outer surface in contact with the water: a gray circular ring appears after about 1 day, and its thickness increases over time due to the penetration of water, which migrates from the edges toward the center of the sample.

It is worth noting that the opaque ring is birefringent since it appears bright when observed through crossed polarizers (Figure 4, central column). The reasons for this birefringence should be further analyzed. It could be attributed to the crazes that cause the opacification of the sample or to small crystallites nucleated on the crazes themselves [22].

**Figure 5:**
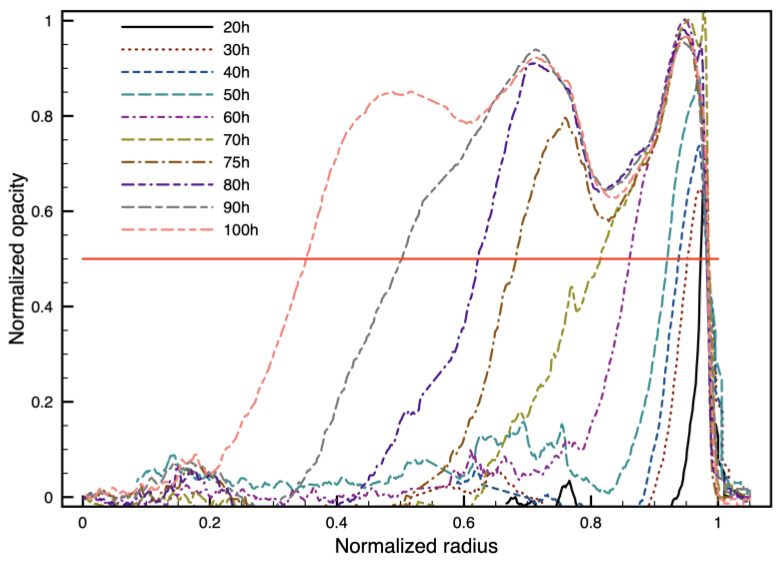
Profile plots of opacity versus the normalized radius (0 = center, 1 = surface in contact with water) at selected times. The threshold level of 50% is reported as a reference.

**Figure 6:**
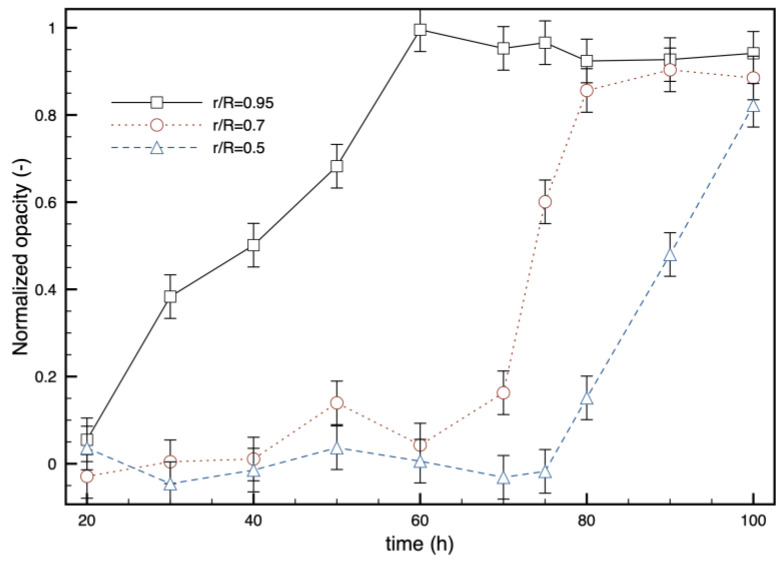
Time evolution of opacity at some radial positions (0 = center, 1 = surface in contact with water) during test A.

The pictures taken during the tests were analyzed using software for image processing (ImageJ—https://imagej.net). After converting each image to grayscale, the software allows plotting the grayscale level along a given path. If the path is a radius, the software provides a measurable profile of the opacity versus the distance from the center of the disk. The results are reported in Figure 5 for a sample under the conditions of test A. The normalization factor is the same for all the profiles, so the values can be directly compared. Since all the samples have the same radius (6.5 mm), the thickness is reported as a fraction of the radius of the PLA disc. The penetration of the opacification towards the center of the sample is evident. In each layer, opacity increases from zero to the maximum value (1, according to the normalization). The profiles are very steep: opacity shifts from 0 to 1 within a space of less than 1 mm before reaching the center of the sample. The time evolution of opacity at some radial positions is reported in Figure 6. The outer ring of the sample becomes opaque within 2.5 days after being in contact with water, when the reduction in number-average molecular weight due to hydrolysis is about 40% [34,35]. After a layer becomes opaque, namely after reaching 1 on the chosen scale, no further increase in opacity with time is detected. Thus, the phenomenon does not seem to be linked directly to hydrolysis since hydrolytic degradation increases with time. As a reference, molecular weight reduction becomes equal to 60% after 100 h at 58 °C, according to the literature [36]. Crystallinity is also expected to increase with time, but this does not seem to cause a further increase in opacity in the time frame of the experiments.

**Figure 7:**
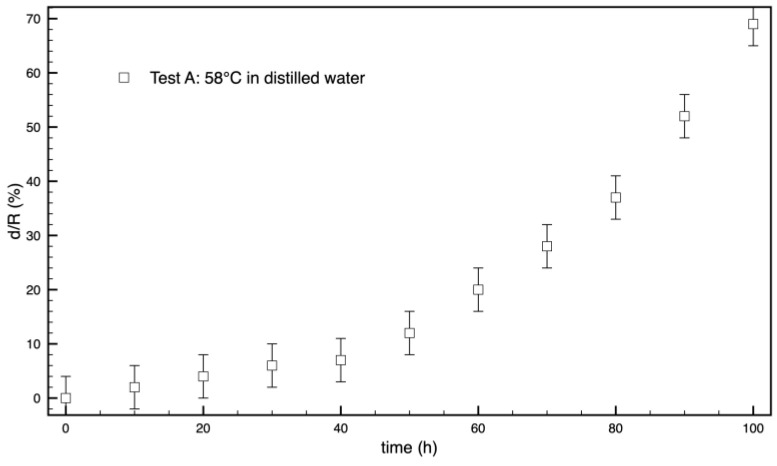
Thickness, *d*, of the opaque ring, normalized with respect to the radius of the sample, versus time. The radius of the sample is 6.5 mm.

By choosing a threshold for the normalized opacity, i.e., 50%, as depicted in Figure 5, it is possible to quantify the thickness of the opaque ring from the profiles. The results are reported in Figure 7. 

The increase in the thickness of the opaque ring versus time, namely the penetration of opacification inside the sample, presents an upward concavity with an exponential or quadratic trend. This means that the phenomenon follows the penetration of water, which instead presents a downward concavity with a dependence on the square root of time, at least in the initial steps.

### 3.2. Samples A, B, and C: Effect of Temperature

**Figure 8:**
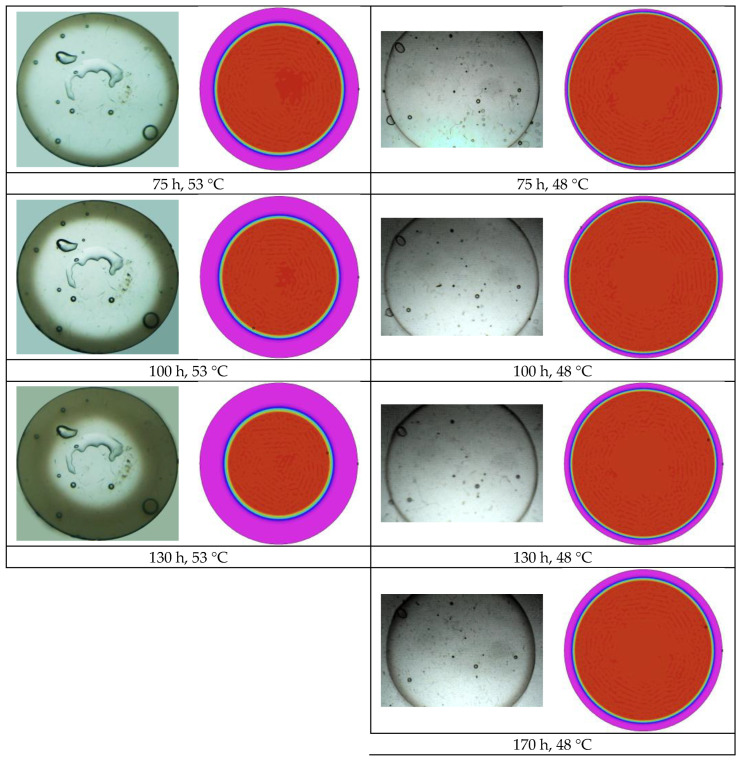
Time evolution of opacification during time for test B, carried out in water at 53 °C, left column, and test C, carried out in water at 48 °C, right column.

The effect of temperature was considered by comparing the results of tests A, B, and C. Some images taken during test B, carried out at 53 °C, and test C, carried out at 48 °C, are reported in Figure 8. In both samples, the opacification process runs slower with respect to test A, and the process is slower as the temperature decreases.

**Figure 9:**
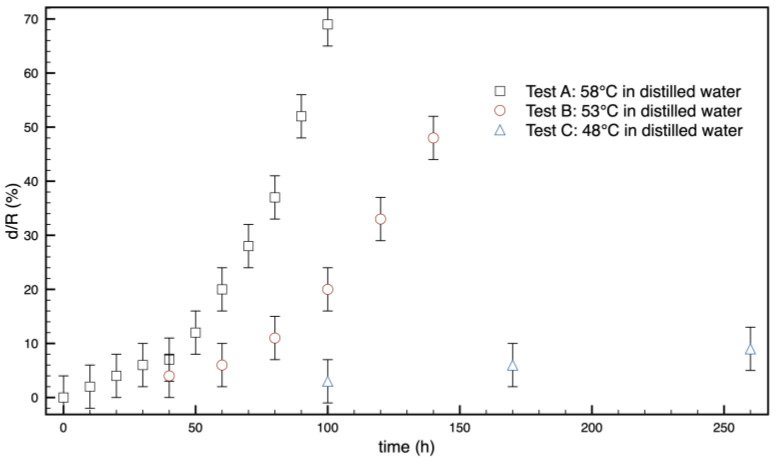
Thickness *d*/*R* of the opaque ring, normalized with respect to the radius of the sample, versus time for tests carried out in distilled water at different temperatures.

A quantification of the phenomenon, conducted by measuring the thickness of the opaque ring, is reported in Figure 9.

It is evident that the opacification time significantly decreases as the temperature increases. This effect could be due to the higher mobility the molecules reach approaching the glass transition.

### 3.3. Samples A, D, and E Effect of Water Concentration

**Figure 10:**
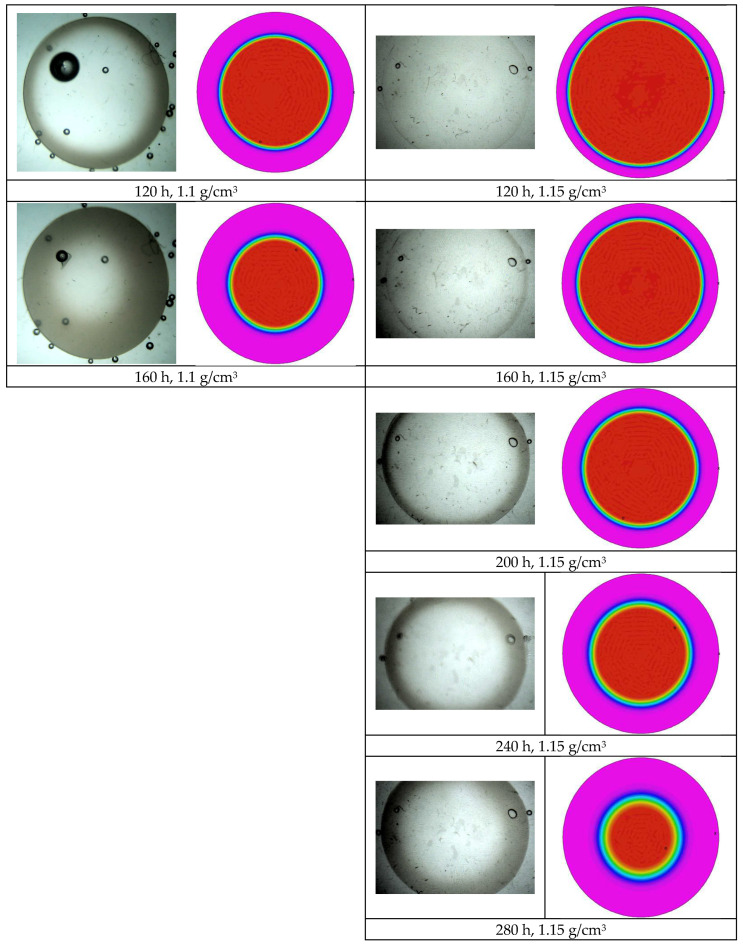
Time evolution of opacification during test D, carried out in a solution with a density of 1.1 g/cm^3^ at 58 °C, left column, and test E, carried out in a solution with a density of 1.15 g/cm^3^ at 58 °C, right column.

The effect of water concentration was considered by comparing the results of tests A, D, and E. Some images taken during tests D, carried out in a solution with a density of 1.1 g/cm^3^, and test E, carried out in a solution with a density of 1.15 g/cm^3^, are reported in Figure 10. In both samples, the opacification process runs slower with respect to test A. The process slows as the solution density increases and, consequently, the equilibrium water content in the solid samples increases as well.

**Figure 11:**
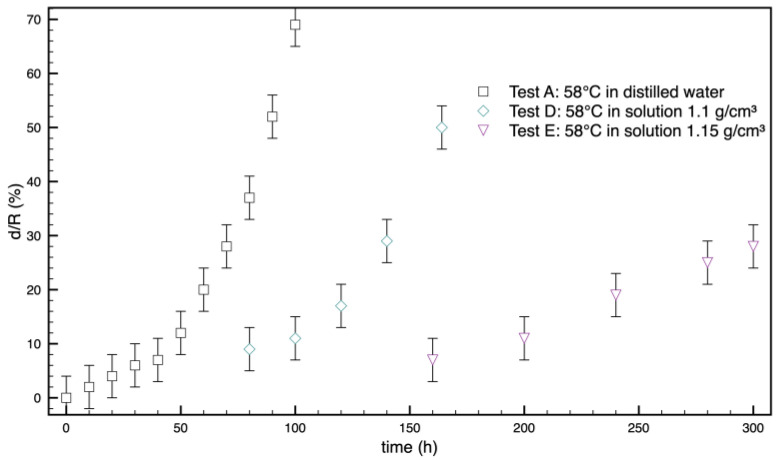
Thickness, d, of the opaque ring normalized with respect to the radius of the sample, versus time for tests carried out in water solutions at different densities at a temperature of 58 °C.

A quantification of the phenomenon, conducted by measuring the thickness of the opaque ring, is reported in Figure 11.

Opacification proceeds faster by decreasing the moisture density.

### 3.4. Analysis of the Results

**Figure 12:**
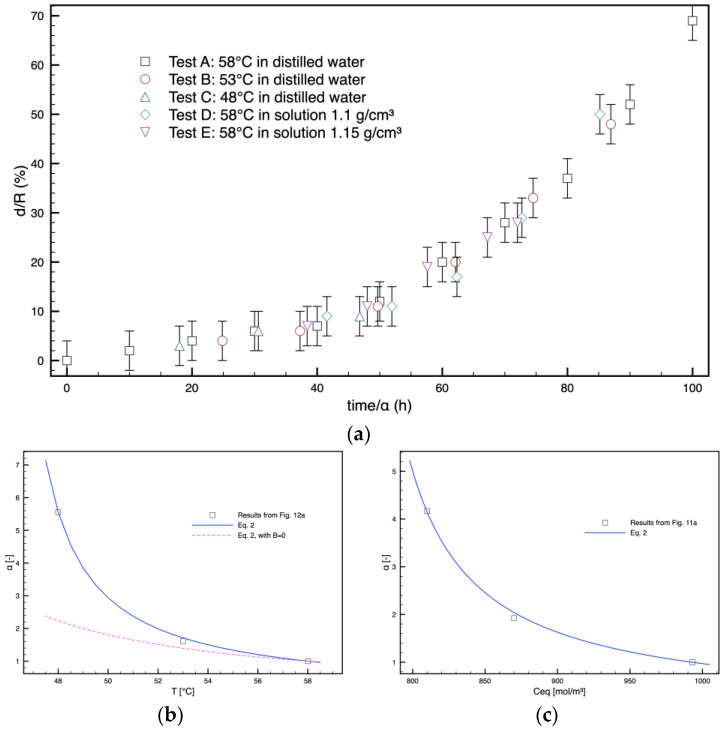
(**a**) Master curve of all the results of thickness of the opaque ring versus time, scaled by a shift factor dependent on temperature (**b**) and equilibrium water concentration (**c**).

A careful observation of Figure 9 and Figure 11 reveals that the shape of the curves describing the evolution of the opaque ring is similar for all the tests. On this basis, an attempt was made to build a master curve of all the results by reporting all the results versus time, scaled by a shift factor α, which was different for each test (and equal to 1 for test A). The results are reported in Figure 12. The results show that on choosing a suitable factor α for each test, all the data collapse on a single curve. The factors α were found to be an increasing function of temperature (Figure 12b) and water concentration at equilibrium, $C_{eq}$ (Figure 12c). 

For tests A, D, and E, the system’s temperature, and thus the water diffusivity inside the solid sample and the hydrolysis kinetics, are the same. Therefore, the only variable changing during the test is the water concentration inside the sample. For samples A, B, and C, the temperature changes, and thus, other relevant parameters, such as diffusivity, hydrolytic degradation, and water concentration, change from test to test. The shift factors are reported in Figure 12b, thereby accounting for different phenomena at the same time.

A fitting equation was considered to describe the effect of temperature and concentration on the shift factor

(2) $$\alpha \left(C_{eq},T\right)=\frac{1}{1+A\left(C_{eq}-C_{ref}\right)+B\left(T-T_{ref}\right)}$$

in which $C_{ref}$ and $T_{ref}$ were set to 993 mol/m^3^ and 331 K (=58 °C). The parameters A and B were identified as 4.2 10^−3^ m^3^/mol and 2.67 10^−2^ K^−1^, respectively.

For the samples in pure water, the following equation was adopted to fit the data reported in Table 1 for tests A, B, and C:

(3) $$C_{eq}\left(T\right)=C_{ref}e^{-\frac{E}{T}+\frac{E}{T_{ref}}}$$

The parameter E was identified as 1530 K, which provides a temperature dependence consistent with the values found in the literature [24]. It is worth mentioning that, despite the fact that much research is available on the water sorption from humid gas, a considerably lower number of papers are available on the water sorption from samples immersed in water [37].

Equations (2) and (3) provide the description reported in Figure 12b,c as lines. In Figure 12b, the effects of temperature on the shift factor are neglected, in order to show that it has a particularly significant impact on the phenomenon. This is particularly evident for tests C and D, which present nearly the same concentration at equilibrium, although the shift factor for test C is close to 5, being much larger than that of test D, which is close to 2. This happens because test C is carried out at a much lower temperature, 48 °C, with respect to test D.

## 4. Modeling and Simulation

The normalization proposed above does not allow for a full description of the phenomenon, since the variable $C_{eq}$ is indeed present in the sample only at the interface with the solution and at equilibrium. Due to diffusion, the water concentration decreases with decreasing radius and increases with time.

With the aim of fully describing the profiles of opacification inside the samples, a simple model was considered. Assuming that the two circular surfaces of the disk can be considered impermeable due to contact with the glass, the water concentration inside the sample is described by the following balance equation:

(4) $$\frac{\partial C}{\partial t}=\frac{1}{r}\cdot \frac{\partial }{\partial r}rD\frac{\partial }{\partial r}C$$

where *C* is the water concentration inside the sample, *D* is the water diffusivity, taken as a function of temperature and opacity, as described below.

The boundary condition at *r = R*, the radius of the disk, was simply *C = C_eq_*, with *C_eq_* depending on the test, according to Table 1.

The opacity evolution was described by the following first-order model:

(5) $$\frac{dOp}{dt}=\frac{Op-{Op}_{\infty }}{\tau \left(C,T\right)}$$

where *Op* is the variable which describes the opacity, whose initial value is 0 for the transparent sample, *Op*_∞_ is the reference value for the fully opaque sample, taken as 1 in this work, and τ is the characteristic time of the phenomenon, whose dependence on temperature and local water concentration was assumed as

(6) $$\tau \left(C,T\right)=\frac{\tau_{ref}}{1+A\left(C-C_{ref}\right)+B\left(T-T_{ref}\right)}$$

The parameters A and B were taken as the same as those identified by Equation (2), whereas the constant $\tau_{ref}$ was used as a best-fitting parameter. It was set to 2 h to obtain the results reported in this work. This value is consistent with the data reported in the literature [22], which showed that the opacification half-time for the thin samples immersed in water reaches its maximum value after about 10 h.

The diffusivity parameter was considered in this work a function of temperature and opacity. The dependence on temperature was taken from the literature [17]. Concerning the effect of opacity, it was considered that the phenomenon is due to crazes induced by water. It is likely that in these crazes water can diffuse much easily than in the compact solid sample. Thus, it was assumed that diffusivity changes linearly with opacity, increasing by a factor equal to 4 when opacity shifts from 0 to 1.

The diffusivity was therefore taken as

(7) $$D\left(T,Op\right)=D_{ref}e^{-\frac{F}{T}+\frac{F}{T_{ref}}}\left(GOp+1\right)$$

with *D_ref_* = 1.5 10^−11^ m^2^/s, *F* = 2942 K, and *G* = 3. Eventually, $\tau_{ref}$ and *G* are the only two adjustable parameters in the model adopted to describe the data.

The model was implemented in a multiphysics software for partial differential equations (flexpde, www.pdesolutions.com), and the results are compared in Figure 4, Figure 8, and Figure 10 with the pictures showing the time evolution of opacity. Despite the simplicity of the model, and the very limited number of free parameters, the main effects are correctly captured. It can be noted that the model predicts faster penetration of opacity at shorter times and slower penetration at longer times in all cases. Further improvement of the model is therefore desirable. The improvement needs, however, further characterization of the model. Important aspects, such as the dependence of water diffusivity on opacity, the swelling effect of water, which affects diffusivity and glass transition, must be characterized and considered in the model. This will be the objective of future work.

## 5. Conclusions

This work analyzed the kinetics of opacification of PLA due to water sorption. Some PLA samples in the form of thin disks were subjected to controlled water sorption tests in radial direction, and the phenomenon of opacification was observed by optical microscopy.

It was shown that over time, a white, opaque ring appears at the maximum radius of the disk and increases its thickness with time. The thickness of the ring was measured at different time intervals, and it was shown that the rate of increase in thickness changes with the test conditions. In particular, on decreasing the temperature and the equilibrium water concentration, the phenomenon slows.

It was shown that the evolution of the thickness of the opaque ring, measured under different conditions of temperature and water equilibrium concentration, can be made to overlap on a single master-curve by adopting suitable time shift factors. The shift factors are constant for each test, and their dependence on temperature and equilibrium water concentration was assessed.

On the basis of the experimental observation, a modeling of the whole phenomenon, including water diffusion and the effect of temperature and equilibrium water concentration, was attempted. The opacity was described by a simple first-order model, with a characteristic time depending on temperature and local water concentration. Although very simple, the model captured the main effects of the variables. A further improvement of the model is desirable, but it can only be reached after further characterization of the phenomena involved, such as the dependence of water diffusivity on opacity.

The description of the opacification kinetics would enable determining the behavior of the PLA parts, mainly the ones used for packaging applications, and predicting more accurately the lifetime of the parts.

## Figures and Tables

**Table 1:** List of the tests carried out in this work.

Test	Temperature (°C)	Solution Density (g/cm^3^)	$C_{eq}$ (mol/m^3^)
A	58 °C	1	993
B	53 °C	1	925
C	48 °C	1	860
D	58 °C	1.1	870
E	58 °C	1.15	810

## Data Availability

Data will be made available upon request.
